# Blood CD3-(CD56 or 16)+ natural killer cell distributions are heterogeneous in healthy adults and suppressed by azathioprine in patients with ANCA-associated vasculitides

**DOI:** 10.1186/s12865-021-00416-w

**Published:** 2021-04-12

**Authors:** Wolfgang Merkt, Ulrich Salzer, Jens Thiel, Ilona Jandova, Raoul Bergner, Ana C. Venhoff, Nils Venhoff

**Affiliations:** 1 Department of Hematology, Oncology and Rheumatology, Internal Medicine V, University Hospital of Heidelberg, Im Neuenheimer Feld 410, 69120 Heidelberg, Germany; 2 Department of Rheumatology and Clinical Immunology, Medical Center, Faculty of Medicine, University of Freiburg, Freiburg im Breisgau, Germany; 3 Department of Rheumatology, Nephrology, Haemato-Oncology, Klinikum Ludwigshafen, Ludwigshafen, Germany

**Keywords:** ANCA-associated vasculitis, Natural killer cells, Azathioprine

## Abstract

**Background:**

Cytotoxic Natural Killer (NK) cells are increasingly recognized as a powerful tool to induce targeted cell death in cancer and autoimmune diseases. Still, basic blood NK cell parameters are poorly defined. The aims of this study were 1) to establish reference values of NK cell counts and percentages in healthy adults; 2) to describe these parameters in the prototype autoimmune disease group ANCA-associated vasculitis (AAV); and 3) to investigate whether NK cell counts and percentages may be used as activity biomarkers in the care of AAV patients, as suggested by a preceding study.

**Methods:**

CD3-(CD56 or 16)+ NK cell counts and percentages were determined in 120 healthy adults. Lymphocyte subset and clinical data from two German vasculitis centers were analyzed retrospectively (in total 407 measurements, including 201/49/157 measurements from 64/16/39 patients with granulomatosis with polyangiitis (GPA), microscopic polyangiitis (MPA) and eosinophilic granulomatosis with polyangiitis (EGPA), respectively).

**Results:**

CD3-(CD56 or 16)+ NK cell counts and percentages in healthy adults were highly variable, not Gaussian distributed and independent of age and sex. NK cell percentages ranged from 1.9 to 37.9% of lymphocytes, and were significantly more dispersed in AAV (0.3 to 57.6%), while the median percentage was not different between AAV and healthy donors. In contrast, median NK cell counts were significantly lower in AAV compared to healthy donors. Sub-group analyses revealed that NK cell counts were low independent of AAV entity and disease activity. Azathioprine therapy was associated with significantly lower NK cell counts and percentages compared to non-azathioprine therapies. In 13.6% of azathioprine-treated patients, percentages were </= 1% which may be interpreted as temporary NK cell deficiency. NK cell counts and percentages could not separate active from inactive AAV.

**Conclusions:**

NK cell counts and percentages in blood are heterogeneous and can presently not be recommended as biomarker in clinical care of AAV patients. Azathioprine treatment was associated with significantly low NK cells. These findings may be relevant for the development of drugs that aim at exploiting NK cell cytotoxicity and may help to identify patients at risk to develop malignant or infectious co-morbidities.

**Supplementary Information:**

The online version contains supplementary material available at 10.1186/s12865-021-00416-w.

## Background

Anti-neutrophil cytoplasmatic antibody (ANCA)-associated vasculitis (AAV) is a rare systemic inflammatory condition affecting mainly small vessels [1, 2]. Despite some overlap, AAV comprise clinically, genetically and pathogenetically different entities. The most common entity is GPA, followed by MPA and EGPA, the latter of which is an especially rare condition. All three diseases are treated depending on activity, severity and the types of organs involved and often necessitate intense immunosuppression, including induction regimens with cyclophosphamide or the B cell-depleting anti-CD20 antibody rituximab [3].

While relatively specific autoantibodies - ANCAs with the two main sub-specificities anti-proteinase 3 (PR3) and anti-myeloperoxidase (MPO) - have some value in taking the diagnoses in AAV, disease activity and course cannot reliably be determined by laboratory tests. Treatment decisions are based on the physicians’ judgments of disease activity and require substantial experience. Therefore, new activity biomarkers are needed for daily care in AAV [4]. In a preceding study, NK cell proportions in blood from patients with GPA were significantly increased in stable remission [5]. The usefulness of NK cells as activity biomarker has not been investigated in other studies, and there are currently no data available on NK cells in MPA and EGPA.

Likewise, the role of NK cells in AAV is poorly understood. Based on their biologically known functions [6], NK cells may theoretically participate in autoimmune systemic inflammation by means of antibody dependent cell cytotoxicity (ADCC), by controlling other immune cells like CD4+ T lymphocytes [7], by secreting cytokines like interferon-ɣ or by killing neighbor cells expressing stress-induced ligands [8]. In our prior studies in GPA, we found that NK cells were mainly mature, functional cells with maintained cytotoxicity receptors, recognition of ligand-expressing target cells and ADCC [5, 9, 10]. However, we also observed several quantitative differences. For example, NK cells showed signs of activation, including the expression of CD69, CD54 and CCR5 and the down-regulation of CD16, in particular in active GPA.

In the last couple of years, NK cells have become increasingly interesting as targets of cancer therapies, as recently summarized in *Nature* [11]. One example is the introduction of new checkpoint inhibitors that target innate immune cells including NK cells which are currently investigated in clinical trials. By virtue of the high expression of CD16 (Fc-ɣ-Receptor IIIa), NK cells can also be targeted via the Fc-part of therapeutic antibodies, which induces ADCC [6, 12]. NK cells thus play a role in the treatment with cell-depleting antibodies [13–16]. Accordingly, rituximab activates NK cells in vivo in patients with AAV [10]. A recent improvement of cell-depleting antibodies was achieved by engineering antibodies with an enhanced affinity to Fc receptors, such as the type 2 anti-CD20 antibody obinutuzumab [17], which is superior to rituximab in the treatment of lymphomas [18]. Obinutuzumab is also a promising treatment strategy in systemic inflammatory diseases and has successfully passed a phase II trial in systemic lupus [19]. We have recently shown that obinutuzumab is highly effective in depleting B cells and activating NK cells within PBMCs from GPA patients in vitro [10]. However, formal proof of whether NK cell-targeting therapies aside rituximab work in systemic inflammatory diseases is pending.

The possible usefulness of NK cells as biomarker in AAV and the increased interest in NK cell-targeting therapies prompted us to investigate NK cells in AAV in more detail. While preparing this study, we frequently found the notion that NK cells comprise “about 5 to 10(or 15 or sometimes 20) percent of peripheral blood lymphocytes”. However, amazingly few state-of-the-art studies sustained these statements. In a study from 2004, “CD3-CD56+ CD16+” NK cells from 51 healthy Caucasian individuals ranged from 51 to 652/μl, corresponding to 2 to 31% of lymphocytes [20]. A similar result was obtained in a representative Swiss cohort of 70 adults in which “CD3−/(CD16+/CD56+)” NK cells ranged from 77 to 427/μl, corresponding to 5.35 to 30.93% of lymphocytes [21]. Notably, the definition of NK cells varies between studies. While many antibody panels used in clinics to determine lymphocyte subsets by flow cytometry rely on commercial kits determining NK cells as “CD3 negative CD16/56 positive”, the consented definition of NK cells among immunologists is “CD3 negative and CD56 positive” [22]. In some studies, it remains unclear whether “CD16/56 positive” means CD16 *or* CD56 positive (e.g., both antibodies are linked to the same fluorochrome) or CD16 *and* CD56 positive (i.e., double-positive, meaning that CD56bright CD16negative NK cells are excluded) [20]. In addition, studies vary in whether other cell types are excluded from the NK cell gate. Today, CD14+ monocytes and CD19+ B cells are usually excluded next to CD3+ T cells prior to gate on CD56+ NK cells [22]. In particular in older studies, NK cells were determined by using only CD16 as single marker [23], or using two-color flow cytometry [21]. Analysis strategies of NK cells may further be variable based on whether they were determined in whole blood or in isolated peripheral blood mononucleated cells (PBMCs) after density gradient centrifugation. Finally, PBMCs can be used either freshly or after a freezing-thawing cycle. These analytical differences may be responsible for the confusion about “normal” NK cell counts and percentages in peripheral blood. We did not find data on the statistical type of distribution of NK cell parameters (e.g. Gaussian). Re-analysis using state-of-the-art multicolor flow cytometry is therefore needed to establish standard ranges and distributions. To this end, we analyzed NK cell data from 120 healthy individuals in the present study. The method we used is a current diagnostic standard in German clinics. With the same protocol, we analyzed blood NK cell counts and percentages from patients with AAV.

## Patients and methods

120 healthy individuals served to establish reference values of CD3-(CD56 or CD16) + NK cell percentages and counts in healthy adults. To describe these parameters in ANCA-associated vasculitis and to test their potential use as disease activity biomarker, we retrospectively analyzed existing lymphocyte subset data from two German vasculitis centers [24]. Between 2011 and 2017 (vasculitis center 1) and 2016 and 2020 (vasculitis center 2), CD3-(CD56 or CD16) + NK cells and matching Birmingham vasculitis activity scores (BVAS) were determined repeatedly from consenting patients that were at least 18 years old and met current ACR classification criteria. In the retrospective analysis, all patients with available data on NK cell parameters were included; there were no exclusion criteria (ethics committee of Freiburg University, file no. 191/11, 46/04). All experiments were performed in accordance with relevant guidelines and regulations. In vasculitis center 1, we analyzed 151/49/157 measurements from 40/16/39 patients with GPA, microscopic polyangiitis (MPA) and eosinophilic granulomatosis with polyangiitis (EGPA), respectively. Unless otherwise stated, all measurements were included in the analyses, i.e. in re-examined patients with more than one flow cytometric determination of NK cell counts and percentages at different time points, all determinations were included. Vasculitis center 2 contributed 50 measurements from 24 GPA patients; these results were analyzed separately (Fig. 6 only). Descriptive parameters are shown in Tables 1 and 2.

**Table 1:** Descriptive statistics: AAV entities, numbers of individuals and counts. HC, healthy control; GPA, granulomatosis with polyangiitis; MPA, microscopic polyangiitis; EGPA, eosinophilic granulomatosis with polyangiitis; BVAS, Birmingham vasculitis activity score; AAV, ANCA-associated vasculitis. “Measurements” refers to existing flow cytometry data on NK cell counts and percentages at a given time point

Vasculitis center	Entity	individuals, n	measurements, n	measurements per individual, mean	measurements per individual, range
1	HC	120	120	1	1
	GPA, total	40	151	3,8	1–8
	_inactive, BVAS =0	40	126	3,2	1–7
	_active, BVAS > 0	15	25	1,7	0–3
	MPA, total	16	49	3,1	1–7
	_inactive, BVAS =0	15	38	2,5	0–6
	_active, BVAS > 0	8	11	1,4	0–3
	EGPA, total	39	157	4,0	1–9
	_inactive, BVAS =0	36	121	3,4	0–8
	_active, BVAS > 0	21	36	1,7	0–4
2	GPA, total	24	50	2,1	1–3
	_inactive, BVAS =0	24	41	1,7	1–3
	_active, BVAS > 0	9	9	1,0	0–1
1 + 2	all patients with AAV	119	407	3,4	nd

**Table 2:** Descriptive statistics: Organ involvement in GPA and sex. GPA, granulomatosis with polyangiitis; MPA microscopic polyangiitis; EGPA, eosinophilic granulomatosis with polyangiitis

Vasculitis center	Entity	Characteristics	Measurements, n	Percentages
1	GPA	systemic	135	89%
		localized	16	11%
		female	84	56%
		male	67	44%
	MPA	female	25	51%
		male	24	49%
	EGPA	female	43	27%
		male	109	69%
		not determined	5	3%
2	GPA	systemic	50	100%
		localized	0	0%
		female	28	56%
		male	22	44%

### Lymphocyte subpopulation phenotyping

**Fig. 1:**
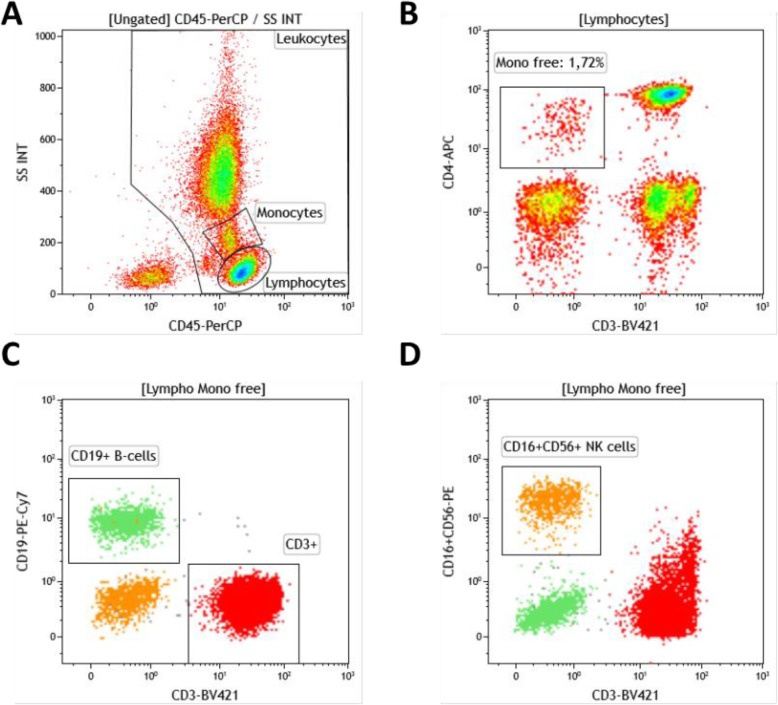
Gating strategy for NK cells in vasculitis center 1. **a** Definition of leucocyte subpopulations of monocytes and lymphocytes by CD45 and sideward scatter (SS). **b** Exclusion of contaminating monocytes in the lymphocyte subpopulation by gating out CD3-CD4dim cells. **c** Identification of CD19+ B-cells and CD3+ T-cells by CD3 versus CD19 staining. **d** Identification CD16+ or CD56 + NK cells by co-staining of CD16 and CD56 versus CD3

Phenotyping of T-, B- and NK cells within the lymphocyte population was performed by a whole blood staining lyse-no wash protocol (Optilyse B, Beckman-Coulter) using six colour flow cytometry with the following fluorochrome-conjugated antibodies: BV421 anti-CD3 (clone UCHT1; Biolegend), APC anti-CD4 (clone SK3;Becton Dickinson), FITC anti-CD8 (clone B9.11; Beckman Coulter Immunotech), PE anti-CD16 (clone 3G8; Beckman Coulter Immunotech), PE-Cy7 anti-CD19 (clone J3–119; Beckman Coulter Immunotech), PerCP anti-CD45 (clone HI30; Biolegend), PE anti-CD56 (clone N901; Beckman Coulter Immunotech). Fixed antibody labelled cells were analyzed within 24 h by flow cytometry (Navios; Beckman Coulter). Absolute cell counts were calculated using a two-platform method with leukocyte and lymphocyte counts determined by a hemocytometer. Flow cytometric data analysis was performed with the help of Kaluza Software 1.5a (Beckman Coulter). A representative gating strategy for definition of analyzed cell populations performed in vasculitis center 1 is described in Fig. 1.

#### Statistical analysis

Exploratory statistical analysis was performed using Graph Pad Prism (versions 5 and 8). *p* values < 0.05 were considered significant and have to be interpreted descriptively. *, **, *** and **** in graphs represent *p* values of < 0.05, < 0.01, < 0.001 and < 0.0001, respectively. Normal distribution was generally not assumed, tests were two-sided and graphs show medians, if not stated otherwise. Mann-Whitney or Kruskal Wallis tests in conjunction with post tests were used to compare two or more groups, respectively. Further specific tests used are indicated in figures or the results text. The strategy to analyze distributions of NK cell parameters (Fig. 3 and supplement) was discussed with and approved by a statistician.

## Results

### Blood CD3-(CD56 or CD16) + NK cell counts and percentages in healthy adults

**Fig. 2:**
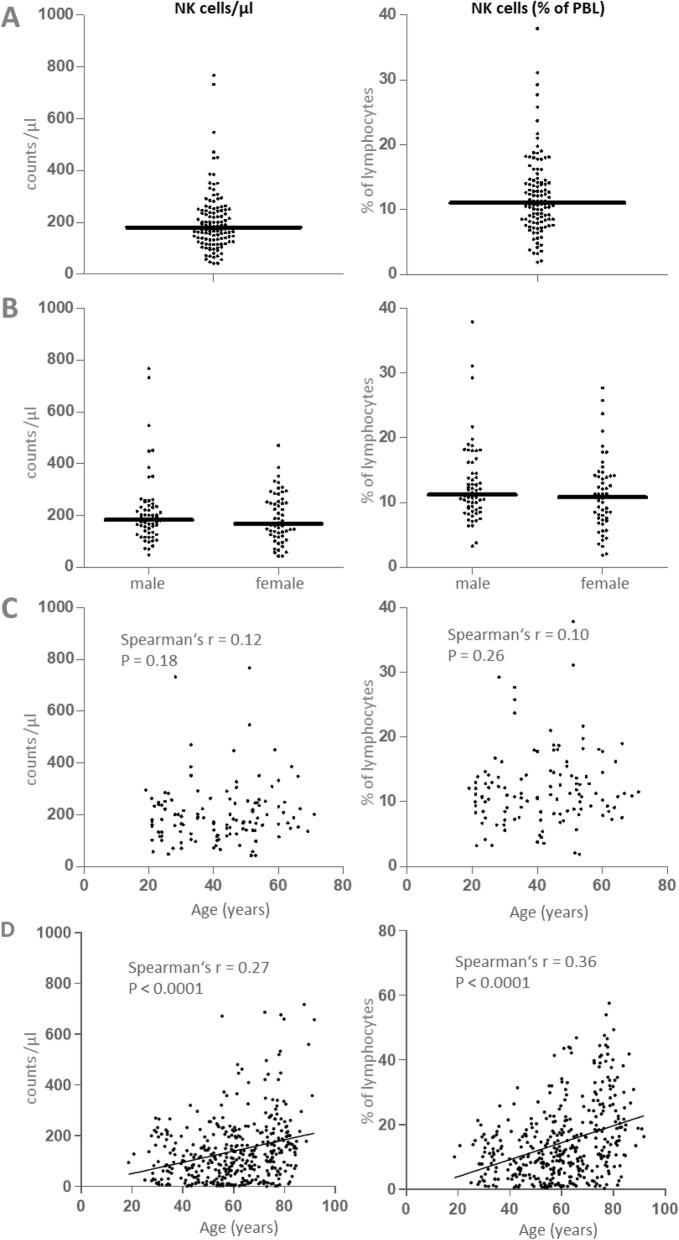
CD3-(CD56 or CD16)+ NK cell counts and percentages in 120 healthy human adults and patients with ANCA-associated vasculitis. **a-c**, data derived from vasculitis center 1. **a**, total healthy cohort. In **b**, healthy men (M, *n* = 63) and women (W, *n* = 57) are plotted separately. In **c** and **d**, counts and percentages are shown in relationship to age in healthy individuals (**c**) and AAV patients (**d**; all patients from both centers are combined)

To establish standard ranges under normal conditions, data from 120 healthy adults were analyzed (Fig. 2). The youngest donor was 19, the oldest 71 years old (median 41.2 years). NK cell counts ranged from 43/μl to 768/μl (median 180.5/μl). Percentages ranged from 1.9 to 37.9% of lymphocytes (median 11.05%) (Fig. 2a). Neither counts nor percentages were Gaussian distributed (D’Agostino & Pearson omnibus normality test). We observed no differences between the two genders (Fig. 2b). Age did not correlate with NK cell counts and percentages (Spearman’s r = 0.12 and 0.10, respectively) (Fig. 2c).

### The distribution of NK cell counts and percentages in ANCA-associated vasculitis

Using the same analysis method, measurements in patients with AAV were performed in vasculitis center 1. Compared to the healthy group, AAV patients were older (18 to 86 years, median 59.5 years; *p* < 0.0001 using Mann-Whitney test) and their NK cell percentages increased with age (Fig. 2d). In Fig. 2d, AAV patients from both vasculitis centers were pooled and the relationship between NK cell parameters and age is shown.

**Fig. 3:**
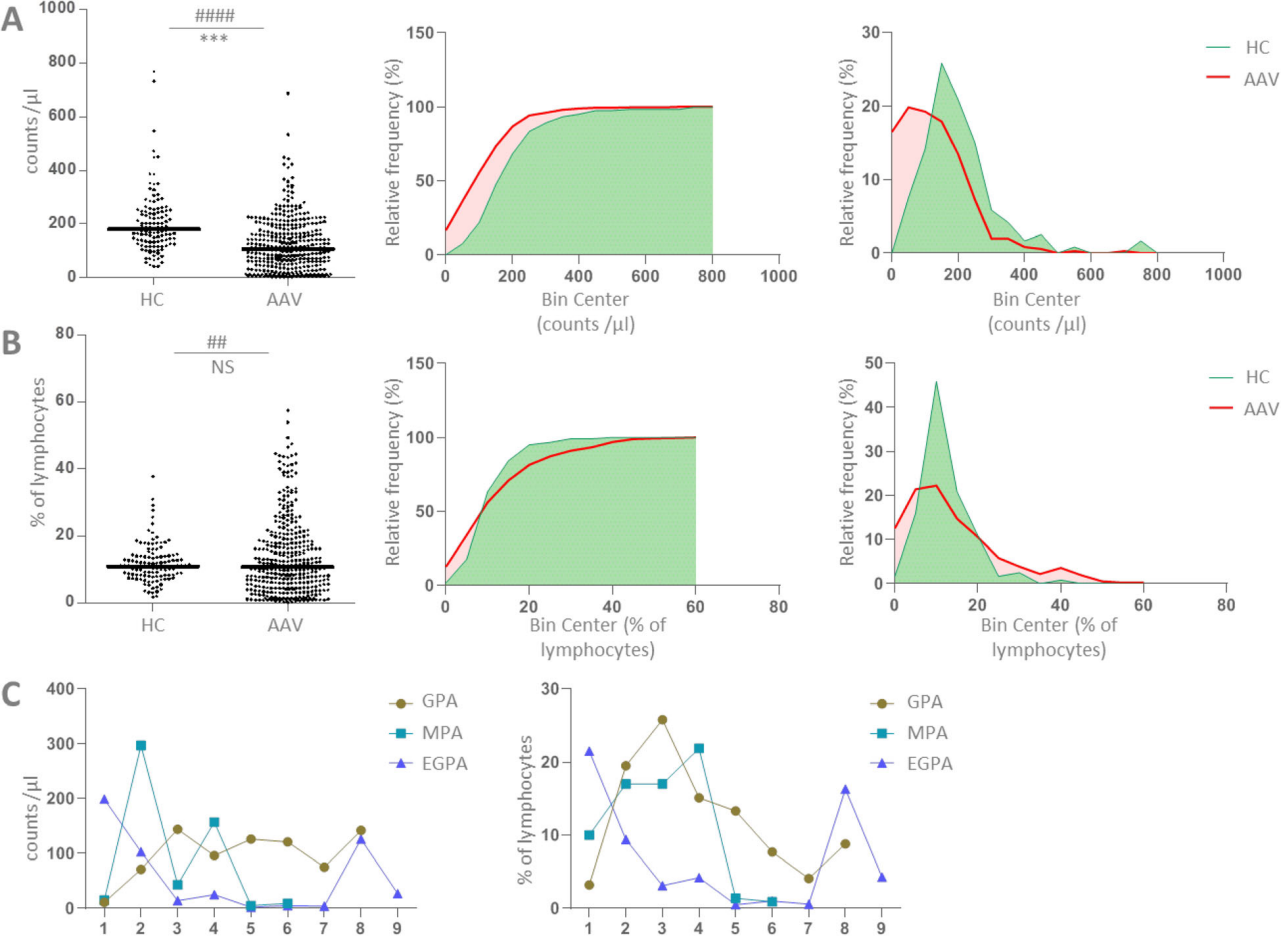
The distribution of NK cell counts and percentages in AAV. **a, b** HC, *n* = 120 healthy controls; AAV, *n* = 357 measurements in 95 AAV patients. The Kolmogorov-Smirnov test revealed different distributions of NK cell counts (**a**) and percentages (**b**) in AAV compared to HC (###, *p* < 0.0001; ##, *p* = 0.0018). The medians were compared using Mann Whitney test (***, *p* < 0.0001; NS, not significant). The middle graphs show the cumulative relative frequencies and the right graphs the actual relative frequencies as histograms, respectively; the bin width was 50 counts/μl and 5%, respectively. In **c**, the course of three patients with GPA, MPA and EGPA over several consecutive measurements (1 to 9) are shown to demonstrate examples of intra-individual variability of NK cell counts (left) and percentages (right). All data derived from vasculitis center 1

Compared to healthy individuals, the distributions of NK cell counts and percentages were different in AAV, as determined by significant Kolmogorov-Smirnov tests and visualized by distribution histograms (Fig. 3). In AAV, NK cell counts ranged from 1/μl to 687/μl (median 106/μl), and percentages ranged from 0.3 to 57.6% of lymphocytes (median 10.8%). The median absolute count was significantly lower in AAV than in HC, and the frequency distribution curves shifted towards low values, accordingly (Fig. 3a). The median percentage of NK cells was not different from HC, but the frequency distribution curves shifted towards relatively more values on both extremes while values around the median were less frequent (Fig. 3b).

One caveat in the analysis shown in Fig. 3 is that all measurements, meaning a variable number of multiple measurements per patients (see Table 1), were included. This could have led to a bias if values in individual patients had been tightly similar. However, we observed a high intra-individual variability in patients with multiple measurements. Figure 3c shows one example patient for each GPA, MPA and EGPA, respectively. It was therefore not possible to pick a single representative value per patient without causing other biases. In an attempt to use one random measurement per patient, we grouped the first and last measurements performed in each patient, respectively, and observed similar distributions as with all measurements (supplementary Fig. 1). Together, these data showed that the distributions of CD3-(CD56 or CD16)+ NK cell counts and percentages in AAV patients were different from that in healthy individuals, with a tendency towards more extreme values.

**Fig. 4:**
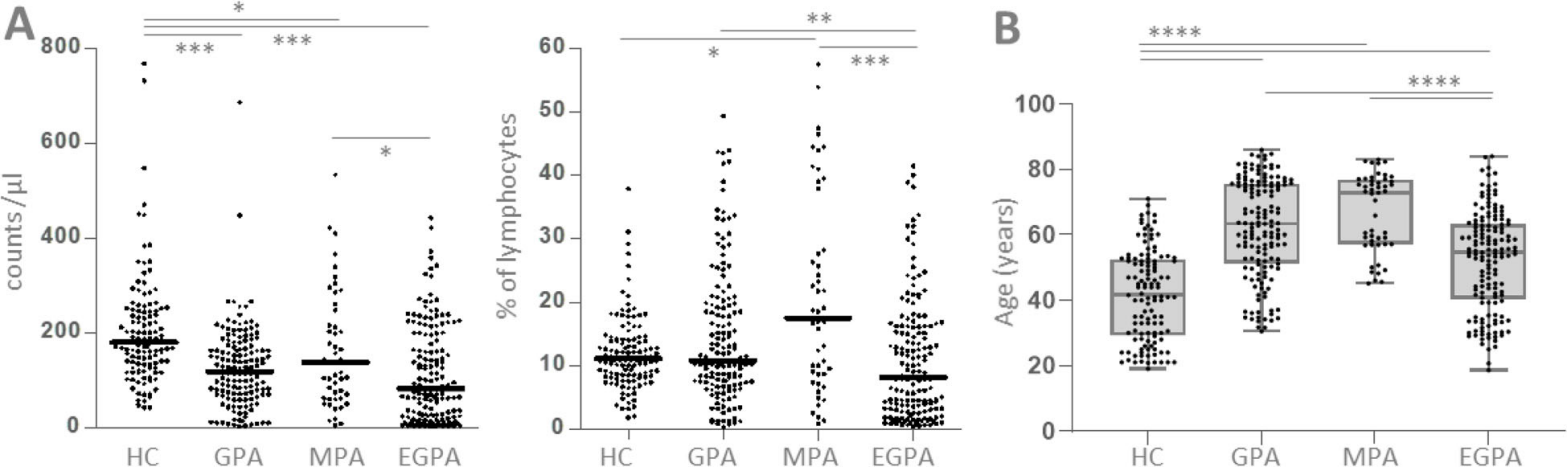
NK cell counts and percentages in GPA, MPA and EGPA. HC, *n =* 120 healthy controls; GPA, *n* = 251 measurements in 40 patients; MPA, *n* = 49 measurements in 16 patients; EGPA, *n* = 157 measurements in 39 patients. **a** NK cell counts and percentages. **b** Age. The Kruskal Wallis test was significant in all graphs (*p* < 0.0001, respectively). Dunn’s post tests were significant where indicated in graphs. Data derived from vasculitis center 1

Next, we analyzed the three AAV entities separately and found that NK cell counts and percentages were significantly different (Fig. 4a). The most prominent differences were the relatively low counts and percentages in EGPA. We observed that 13 measurements (8.2%) in EGPA corresponding to 7 different patients (18%) revealed a percentage of 1% of lymphocytes or below. As we observed weak correlations of NK cell counts and percentages with age in AAV (Fig. 2d) and differences of these parameters between AAV entities, we compared the age between HC and AAV entities. EGPA patients were younger than GPA and MPA patients, but older then HC (Fig. 4b). Therefore, age discrepancies alone did not explain low NK cell counts and percentages in EGPA.

### NK cells in relationship to AAV activity

A previous study found that NK cell percentages may be used as disease activity biomarker in the most common form of AAV, granulomatosis with polyangiitis (GPA) [5]. In order to control this finding, we defined AAV activity based on Birmingham vasculitis activity scores (BVAS). BVAS = 0 defined inactive and BVAS> 0 active disease. BVAS reflects the presence of clinical symptoms and is therefore relevant for treatment decisions.

After sorting the data from vasculitis center 1 according to AAV entity and activity, we found that NK cell counts were

**Fig. 5:**
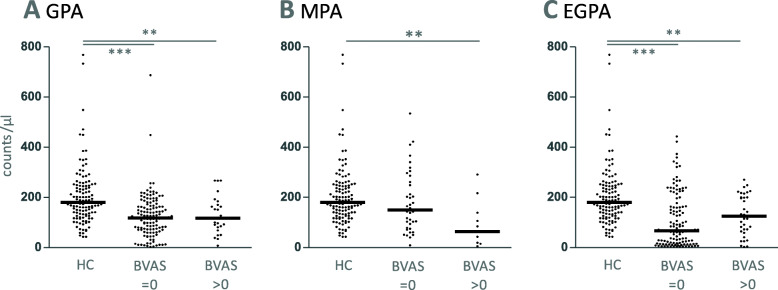
NK cell counts in relationship to disease activity. **a** granulomatosis with polyangiitis (GPA), **b** microscopic polyangiitis (MPA) and **c** eosinophilic granulomatosis with polyangiitis (EGPA). The Birmingham Vasculitis Activity Score (BVAS) was used to differentiate active (> 0) and inactive (=0) AAVs. An exploratory statistical analysis was performed using Kruskal-Wallis test (*p <* 0.0001; =0.0023; < 0.0001 in GPA; MPA; EGPA, respectively). Dunn’s post tests were significant as indicated by stars. All data derived from vasculitis center 1

- significantly lower in all active AAVs compared to healthy controls,
- significantly lower in inactive GPA and EGPA compared to healthy controls,
- not significantly different between active and inactive AAVs (Fig. 5).

NK cell percentages were

**Fig. 6:**
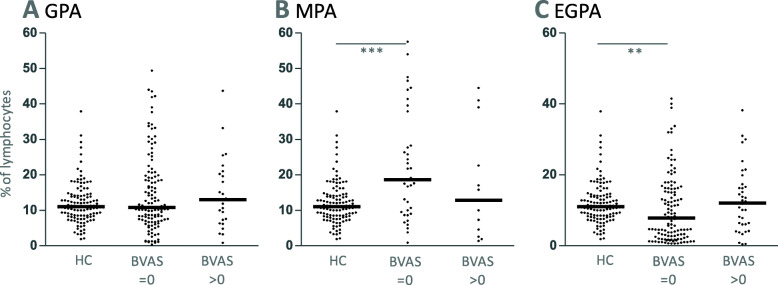
NK cell percentages in relationship to AAV activity. **a** GPA, **b** MPA and **c** EGPA. Kruskal-Wallis tests were not significant in the case of GPA, but significant in MPA and EGPA (*p* = 0.0012 and *p* = 0.0020, respectively). Dunn’s post tests were significant where indicated. Data derived from vasculitis center 1

- significantly higher in inactive MPA compared to healthy controls,
- significantly lower in inactive EGPA compared to healthy controls,
- not significantly different between active and inactive AAVs (Fig. 6).

In EGPA, both counts and percentages were decreased compared to healthy controls, especially in inactive disease (Figs. 5c and 6c). We did not observe any correlation between CRP or anti-Proteinase 3 autoantibody concentration and NK cell counts or percentages (Spearman’s r < 0.3, respectively).

**Fig. 7:**
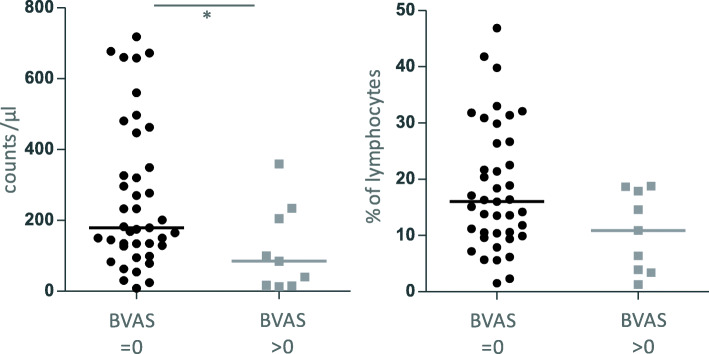
NK cell counts and percentages from GPA patients from vasculitis center 2. BVAS = 0, *n* = 41; BVAS> 0, *n* = 9. Statistical analysis was performed using Mann Whitney test (*, *p* < 0.05). All data derived from vasculitis center 2

In contrast to the previous study [5], we did not observe a difference between active and inactive GPA in vasculitis center 1. As the definition and gating strategy of NK cells varied between the two studies, we next examined data from GPA patients from another vasculitis center (center 2 in the present study, Tables 1 and 2), which used a similar gating strategy to that used in vasculitis center 1. Similar to the data from vasculitis center 1, NK cell counts and percentages were highly variable in GPA patients from vasculitis center 2 and ranged from 8/μl to 718/μl (median 171.5/μl) and from 1.3 to 46.9% (median 14.85%), respectively (Fig. 7). NK cell counts were significantly higher in inactive GPA. Percentages tended to be increased in inactive GPA, without reaching significance. The median age from GPA patients from vasculitis center 2 was 70.3 years and ranged from 27.7 to 91.8 years, which was not statistically different from GPA patients from center 1.

#### NK cells in relationship to therapeutic agents

**Fig. 8:**
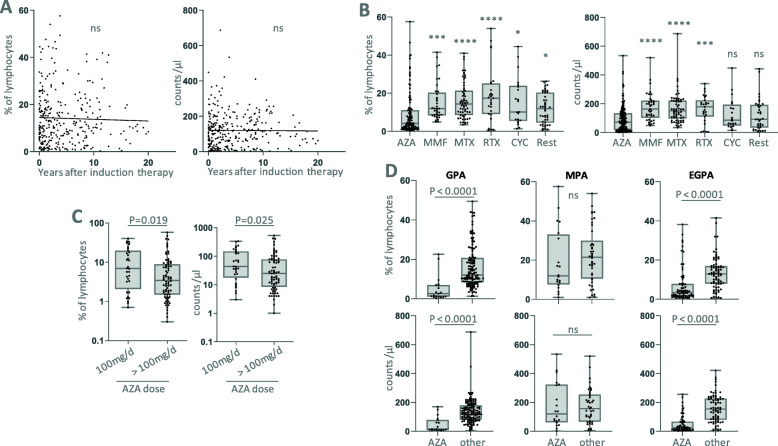
NK cells in relationship to therapeutic agents. Measurements in AAV from vasculitis center 1. **a** NK cell parameters depending on the period of time after induction therapy. **b** NK cell percentages and counts were separated according to drug intake. AZA, azathioprine (*n* = 110 measurements); MMF, mycophenolate mofetil (*n* = 37); MTX, methotrexate (*n* = 62); RTX, rituximab (*n* = 27); CYC, cyclophosphamide (*n* = 21, including three patients with concomitant RTX therapy); other (*n* = 46), including not determined (*n* = 19), no immunosuppressive therapy (*n* = 5), treatment naïve (*n* = 10) and prednisone monotherapy (*n =* 9, from 2.5 to 10 mg, mean 5 mg daily). Kruskal Wallis tests confirmed significance (*p <* 0.0001), the stars indicate significant Dunn’s post tests with azathioprine as reference. **c** Measurements under azathioprine therapy separated according to dosages of 100 mg or > 100 mg daily (*n* = 32 and *n* = 76, respectively). In two patients the dosage was not determinable. Note the logarithmic scale on the y axes. **d** Comparison of azathioprine or other drug intake depending on AAV entity. AZA, *n =* 19, 20, 68; other drugs, *n* = 133, 42, 79 for GPA, MPA and EGPA, respectively. Statistical analysis was performed in (**c**) and (**d**) using Mann Whitney test. ns, not significant

Next to disease subtype and activity, therapeutic agents may alter lymphocyte subsets. In vasculitis center 1, we found no correlation between NK cell counts and percentages with the time span after induction therapy. This indicates that there is no major impact of whether a patient is treated for remission induction (usually high dose glucocorticoids combined with cyclophosphamide or rituximab) or for maintenance of remission (Fig. 8a). When sorting data according to treatments during data acquisition, we found that azathioprine was associated with significantly lower NK cell percentages and counts compared to other drugs (Fig. 8b-d). Higher azathioprine dosages were associated with lower NK cell values, when using a threshold of 100 mg daily (Fig. 8c), suggesting a dose-dependent effect of azathioprine on NK cell parameters. In 15 of 110 (13.6%) measurements during azathioprine therapy, NK cells were below or equal to 1% of lymphocytes.

With the available retrospective dataset, we were not able to perform correlations with the daily or cumulative dose of prednisone.

In vasculitis center 2, no patient received azathioprine at the time of blood donation, all patients were treated with rituximab.

## Discussion

Given the paucity of state-of-the-art studies describing NK cell counts and percentages under normal conditions, we analyzed data from 120 healthy adults. To the best of our knowledge, this is the largest study on normal NK cell counts and percentages so far. Another study comprising 253 healthy people counted 82 to 594 NK cells/μl blood (mean 253/μl), but percentages among lymphocytes were not provided [25]. We did not find differences dependent on age or gender within this adult cohort with a maximum age of 71 years. Our data instead show a heterogeneous distribution and variability of NK cells in blood, and add the information that NK cells are non-normally distributed.

This NK cell variability was further increased in patients with inflammatory disease. In AAV, the frequency distributions of NK cell counts were shifted towards more extremely low values, and the frequency distribution of NK cell percentages was shifted towards both extremes. Accordingly, very high and very low percentages can be encountered in patients with AAV. This circumstance should be considered when planning trials with therapeutic antibodies that bind to FcɣRIIIA with high affinity (such as obinutuzumab), or when established NK cell-targeting therapeutics are administered in order to treat cancers in patients with systemic inflammatory diseases. So far, it is unknown whether NK cell counts or percentages affect the outcome of NK cell-based therapeutics or cancer incidence.

In contrast to healthy individuals, we found a weak, yet significant correlation of NK cell percentages and age in AAV patients; relatively numerous NK cells were found in very old patients with an age > 70 years. This finding is in line with the statement of two reviews concluding that NK cell percentages and counts are increased in the elderly [26, 27]. However, it needs to be noted that, if present, correlations were overall weak or borderline and differences between age groups were small [28, 29]. In addition, some studies compared very young to very old individuals (e.g., 21–30 years vs. 74–96 years) [29, 30]. Therefore, even though there is a chance that NK cell percentages and possibly counts may be increased in the elderly, the variability of these parameters – as described in the present study – rules out conclusions from age on NK cell parameters.

Another aim of the study was to describe and compare NK cell parameters in all three entities of AAV. No data on NK cell counts and percentages were available in the literature for MPA and EGPA so far. The type of retrospective analysis of data from AAV patients we used has several limitations. One is the diverging numbers of measurements per patient. Individual patients might be over- or under-represented. However, NK cell parameters were often variable within individual patients, so that it was impossible to pick out one measurement per patient without causing biases. The strengths of this study lie in the huge number of measurements - it is the largest published cohort on NK cell counts and percentages in ANCA-associated vasculitides - and in its independency from study conditions. Based on our data, we conclude that NK cells are differently distributed in AAV compared to healthy adults, with a tendency towards more extreme values.

The most striking finding of the present study is the association between azathioprine therapy and low blood NK cells. The data suggest that azathioprine significantly influences the NK cell compartment, possibly in a dose-dependent manner. Low NK cell counts during azathioprine therapy were previously reported in patients with inflammatory bowel disease [31, 32].

Some AAV patients had NK cell percentages below 1%, which may be interpreted as a secondary, numerical NK cell deficiency in analogy to the definition of primary NK cell deficiency. The latter is defined by the inherited persistent lack of NK cells (< 1%) in absence of other NK cytopenia-inducing factors (and alternatively by the lack of NK cell cytotoxicity against K562 target cells) [33, 34]. Symptoms occur in childhood and are mainly caused by Herpes virus infections. Whether low NK cells predispose to viral infections and/or tumors in AAV has not been investigated. However, in our previous study, we showed that NK cell percentages correlated with the cytotoxicity against K562 target cells [9], indicating that extremely low NK cells may be associated with a reduced clearance of NK cell targets.

The relevance of low NK cells during azathioprine therapy remains undefined; however, it may be interesting to investigate in the future whether the reduction of systemic NK cells contributes to the therapeutic effect of this immunosuppressive drug. Even though not yet formally proven in AAV, NK cells can be regarded as the extended arm of an adaptive, antibody-mediated immune response by virtue of their Fc-receptor CD16. NK cells and antibody-producing B cells/plasma cells form a functional group, with the common goal to eliminate antigens or pathogenic cells [6, 10]. While B cell depletion with rituximab is a highly efficient treatment in AAV patients, strategies to deplete Fc-receptor bearing cells such as NK cells have so far not been in the focus of clinical researchers.

Based on their physiologic functions as a first line barrier against malignancies and infectious diseases [6], especially in the defense against herpes viruses [33], low NK cells may be a risk factor for malignant diseases or viral infections. Increased cancer risk in post-transplant and possibly inflammatory disease patients treated with azathioprine is a clinically relevant concern [35–37]. A randomized controlled trial confirmed that azathioprine increases the risk to develop warts and herpes simplex infections in patients with inflammatory bowel disease [38, 39]. It remains to be investigated whether these azathioprine-intrinsic risks are in part due to low blood NK cells, and whether low NK cells in AAV patients treated with azathioprine are clinically relevant.

The third aim of the present study was to find out whether NK cell percentages and/or counts can be used to distinguish active from inactive GPA. While recommendations prefer a strict definition of “remission” in clinical trials - requiring a detailed evaluation of the patient (> 6 months absence of symptoms, absence of increased immunosuppression, low and stable doses of prednisone) - [40], we here used an approach based solely on the presence or absence of symptoms (BVAS = 0 vs. BVAS> 0). This approach was supposed to have the highest clinical practicability, especially with respect to treatment decisions which are based on the actual presence of symptoms. In contrast to the previous study [5], in vasculitis center 1, NK cell counts and percentages were not significantly different between active and inactive disease (BVAS > 0 vs. BVAS =0). However, in vasculitis center 2, we observed higher counts in inactive GPA, a finding similar to the previous study [5]. These discrepancies in GPA may be explained by several factors, including different definitions and gating strategies of NK cells between the pilot study and the present study, different study populations between the vasculitis centers, possible regional treatment habits, diverging immunosuppressive therapy, and different clinicians judging activity (BVAS), but also the fewer measurements in center 2. It is also known that environmental factors have an impact on AAV [41]. However, we explicitly waived these factors in the present evaluation of an association between NK cell parameters and AAV activity, because these factors are, in their complexity, impossible to be evaluated in retrospective analyses. The results therefore allow the statement that in routine clinical care, i.e. in an undefined cohort as met by treating physicians, CD3-(CD56 or 16)+ NK cells are currently not helpful to exclude or include disease activity. Noteworthy, the data presented here do not exclude a potential utility of other NK cell parameters like the expression of surface molecules as biomarkers [9].

## Conclusion

In conclusion, NK cells are highly variable in healthy adults, especially in patients with systemic inflammatory disease like AAV. Particularly low NK cell parameters in azathioprine-treated patients warrant further clinical and pathophysiological evaluation. Despite differences compared to healthy controls, CD3-(CD56 or 16)+ NK cell counts and percentages in AAV patients can presently not be recommended as disease activity marker in “real life” clinical practice.

## Supplementary Information

**Additional file 1: Supplementary Fig. 1.** The distribution of NK cell counts and percentages in AAV (one measurement/patient). HC, *n* = 120 healthy controls; Upper left, *n* = 93 first measurements of absolute NK cell counts in patients with ANCA-associated vasculitis (AAV). Upper right, *n* = 95 first measurements of NK cell percentages in blood lymphocytes. Lower row, *n* = 94 last measurements of NK counts and percentages, respectively. In each graph of this figure, the Kolmogorov-Smirnov tests were significant. Data derived from vasculitis center 1.

## Data Availability

The datasets supporting the conclusions of this article are presented within the article and its additional file(s). Further datasets analyzed during the study are available from the corresponding authors on reasonable request.

## References

[1] Cornec D, Gall ECL, Fervenza FC, Specks U (2016). ANCA-associated vasculitis - clinical utility of using ANCA specificity to classify patients. Nat Rev Rheumatol.

[2] Kallenberg CG (2014). Key advances in the clinical approach to ANCA-associated vasculitis. Nat Rev Rheumatol.

[3] Stone JH, Merkel PA, Spiera R, Seo P, Langford CA, Hoffman GS, Kallenberg CG, St Clair EW, Turkiewicz A, Tchao NK, Webber L, Ding L, Sejismundo LP, Mieras K, Weitzenkamp D, Ikle D, Seyfert-Margolis V, Mueller M, Brunetta P, Allen NB, Fervenza FC, Geetha D, Keogh KA, Kissin EY, Monach PA, Peikert T, Stegeman C, Ytterberg SR, Specks U, RAVE-ITN Research Group (2010). Rituximab versus cyclophosphamide for ANCA-associated vasculitis. N Engl J Med.

[4] Monach PA (2014). Biomarkers in vasculitis. Curr Opin Rheumatol.

[5] Merkt W, Sturm P, Lasitschka F, Tretter T, Watzl C, Saure D, Hundemer M, Schwenger V, Blank N, Lorenz HM, Cerwenka A (2015). Peripheral blood natural killer cell percentages in granulomatosis with polyangiitis correlate with disease inactivity and stage. Arthritis Res Ther.

[6] Vivier E, Tomasello E, Baratin M, Walzer T, Ugolini S (2008). Functions of natural killer cells. Nat Immunol.

[7] Cerboni C, Zingoni A, Cippitelli M, Piccoli M, Frati L, Santoni A (2007). Antigen-activated human T lymphocytes express cell-surface NKG2D ligands via an ATM/ATR-dependent mechanism and become susceptible to autologous NK- cell lysis. Blood.

[8] Chan CJ, Smyth MJ, Martinet L (2014). Molecular mechanisms of natural killer cell activation in response to cellular stress. Cell Death Differ.

[9] Merkt W, Claus M, Blank N, Hundemer M, Cerwenka A, Lorenz HM, Watzl C (2016). Active but not inactive granulomatosis with polyangiitis is associated with decreased and phenotypically and functionally altered CD56(dim) natural killer cells. Arthritis Res Ther.

[10] Urlaub D, Zhao S, Blank N, Bergner R, Claus M, Tretter T, Lorenz HM, Watzl C, Merkt W (2019). Activation of natural killer cells by rituximab in granulomatosis with polyangiitis. Arthritis Res Ther.

[11] Demaria O, Cornen S, Daëron M, Morel Y, Medzhitov R, Vivier E (2019). Harnessing innate immunity in cancer therapy. Nature.

[12] Shimasaki N, Jain A, Campana D (2020). NK cells for cancer immunotherapy. Nat Rev Drug Discov.

[13] Veeramani S, Wang SY, Dahle C, Blackwell S, Jacobus L, Knutson T, Button A, Link BK, Weiner GJ (2011). Rituximab infusion induces NK activation in lymphoma patients with the high-affinity CD16 polymorphism. Blood.

[14] Rudnicka D, Oszmiana A, Finch DK, Strickland I, Schofield DJ, Lowe DC, Sleeman MA, Davis DM (2013). Rituximab causes a polarization of B cells that augments its therapeutic function in NK-cell-mediated antibody-dependent cellular cytotoxicity. Blood.

[15] Herndler-Brandstetter D, Shan L, Yao Y, Stecher C, Plajer V, Lietzenmayer M, Strowig T, de Zoete MR, Palm NW, Chen J, Blish CA, Frleta D, Gurer C, Macdonald LE, Murphy AJ, Yancopoulos GD, Montgomery RR, Flavell RA (2017). Humanized mouse model supports development, function, and tissue residency of human natural killer cells. Proc Natl Acad Sci U S A.

[16] Merkt W, Lorenz HM, Watzl C (2016). Rituximab induces phenotypical and functional changes of NK cells in a non-malignant experimental setting. Arthritis Res Ther.

[17] Mossner E (2010). Increasing the efficacy of CD20 antibody therapy through the engineering of a new type II anti-CD20 antibody with enhanced direct and immune effector cell-mediated B-cell cytotoxicity. Blood.

[18] Goede V, Fischer K, Busch R, Engelke A, Eichhorst B, Wendtner CM, Chagorova T, de la Serna J, Dilhuydy MS, Illmer T, Opat S, Owen CJ, Samoylova O, Kreuzer KA, Stilgenbauer S, Döhner H, Langerak AW, Ritgen M, Kneba M, Asikanius E, Humphrey K, Wenger M, Hallek M (2014). Obinutuzumab plus chlorambucil in patients with CLL and coexisting conditions. N Engl J Med.

[19] Furie RAG, A. A, Fragoso-Loyo H, Zuta Santillán E, Rovin B, Schindler T, et al. A Phase II Randomized, Double-Blind, Placebo-Controlled Study to Evaluate the Efficacy and Safety of Obinutuzumab or Placebo in Combination with Mycophenolate Mofetil in Patients with Active Class III or IV Lupus Nephritis [abstract]. Arthritis Rheum. 2019;71(suppl 10).

[20] Pascal V, Schleinitz N, Brunet C, Ravet S, Bonnet E, Lafarge X, Touinssi M, Reviron D, Viallard J F, Moreau J F, Déchanet-Merville J, Blanco P, Harlé J R, Sampol J, Vivier E, Dignat-George F, Paul P (2004). Comparative analysis of NK cell subset distribution in normal and lymphoproliferative disease of granular lymphocyte conditions. Eur J Immunol.

[21] Bisset LR, Lung TL, Kaelin M, Ludwig E, Dubs RW (2004). Reference values for peripheral blood lymphocyte phenotypes applicable to the healthy adult population in Switzerland. Eur J Haematol.

[22] Cossarizza A, Chang HD, Radbruch A, Akdis M, Andrä I, Annunziato F, Bacher P, Barnaba V, Battistini L, Bauer WM, Baumgart S, Becher B, Beisker W, Berek C, Blanco A, Borsellino G, Boulais PE, Brinkman RR, Büscher M, Busch DH, Bushnell TP, Cao X, Cavani A, Chattopadhyay PK, Cheng Q, Chow S, Clerici M, Cooke A, Cosma A, Cosmi L, Cumano A, Dang VD, Davies D, Biasi S, del Zotto G, Della Bella S, Dellabona P, Deniz G, Dessing M, Diefenbach A, di Santo J, Dieli F, Dolf A, Donnenberg VS, Dörner T, Ehrhardt GRA, Endl E, Engel P, Engelhardt B, Esser C, Everts B, Dreher A, Falk CS, Fehniger TA, Filby A, Fillatreau S, Follo M, Förster I, Foster J, Foulds GA, Frenette PS, Galbraith D, Garbi N, García-Godoy MD, Geginat J, Ghoreschi K, Gibellini L, Goettlinger C, Goodyear CS, Gori A, Grogan J, Gross M, Grützkau A, Grummitt D, Hahn J, Hammer Q, Hauser AE, Haviland DL, Hedley D, Herrera G, Herrmann M, Hiepe F, Holland T, Hombrink P, Houston JP, Hoyer BF, Huang B, Hunter CA, Iannone A, Jäck HM, Jávega B, Jonjic S, Juelke K, Jung S, Kaiser T, Kalina T, Keller B, Khan S, Kienhöfer D, Kroneis T, Kunkel D, Kurts C, Kvistborg P, Lannigan J, Lantz O, Larbi A, LeibundGut-Landmann S, Leipold MD, Levings MK, Litwin V, Liu Y, Lohoff M, Lombardi G, Lopez L, Lovett-Racke A, Lubberts E, Ludewig B, Lugli E, Maecker HT, Martrus G, Matarese G, Maueröder C, McGrath M, McInnes I, Mei HE, Melchers F, Melzer S, Mielenz D, Mills K, Mirrer D, Mjösberg J, Moore J, Moran B, Moretta A, Moretta L, Mosmann TR, Müller S, Müller W, Münz C, Multhoff G, Munoz LE, Murphy KM, Nakayama T, Nasi M, Neudörfl C, Nolan J, Nourshargh S, O'Connor JE, Ouyang W, Oxenius A, Palankar R, Panse I, Peterson P, Peth C, Petriz J, Philips D, Pickl W, Piconese S, Pinti M, Pockley AG, Podolska MJ, Pucillo C, Quataert SA, Radstake TRDJ, Rajwa B, Rebhahn JA, Recktenwald D, Remmerswaal EBM, Rezvani K, Rico LG, Robinson JP, Romagnani C, Rubartelli A, Ruckert B, Ruland J, Sakaguchi S, Sala-de-Oyanguren F, Samstag Y, Sanderson S, Sawitzki B, Scheffold A, Schiemann M, Schildberg F, Schimisky E, Schmid SA, Schmitt S, Schober K, Schüler T, Schulz AR, Schumacher T, Scotta C, Shankey TV, Shemer A, Simon AK, Spidlen J, Stall AM, Stark R, Stehle C, Stein M, Steinmetz T, Stockinger H, Takahama Y, Tarnok A, Tian ZG, Toldi G, Tornack J, Traggiai E, Trotter J, Ulrich H, der Braber M, Lier RAW, Veldhoen M, Vento-Asturias S, Vieira P, Voehringer D, Volk HD, Volkmann K, Waisman A, Walker R, Ward MD, Warnatz K, Warth S, Watson JV, Watzl C, Wegener L, Wiedemann A, Wienands J, Willimsky G, Wing J, Wurst P, Yu L, Yue A, Zhang Q, Zhao Y, Ziegler S, Zimmermann J (2017). Guidelines for the use of flow cytometry and cell sorting in immunological studies. Eur J Immunol.

[23] Tollerud DJ, Clark JW, Brown LM, Neuland CY, Pankiw-Trost LK, Blattner WA, Hoover RN (1989). The influence of age, race, and gender on peripheral blood mononuclear-cell subsets in healthy nonsmokers. J Clin Immunol.

[24] Thiel J, Rizzi M, Engesser M, Dufner AK, Troilo A, Lorenzetti R, Voll RE, Venhoff N (2017). B cell repopulation kinetics after rituximab treatment in ANCA-associated vasculitides compared to rheumatoid arthritis, and connective tissue diseases: a longitudinal observational study on 120 patients. Arthritis Res Ther.

[25] Apoil PA, Puissant-Lubrano B, Congy-Jolivet N, Peres M, Tkaczuk J, Roubinet F, Blancher A (2017). Reference values for T, B and NK human lymphocyte subpopulations in adults. Data Brief.

[26] Hazeldine J, Lord JM (2013). The impact of ageing on natural killer cell function and potential consequences for health in older adults. Ageing Res Rev.

[27] Plackett TP, Boehmer ED, Faunce DE, Kovacs EJ (2004). Aging and innate immune cells. J Leukoc Biol.

[28] Le Garff-Tavernier M (2010). Human NK cells display major phenotypic and functional changes over the life span. Aging Cell.

[29] Lutz CT, Karapetyan A, al-Attar A, Shelton BJ, Holt KJ, Tucker JH, Presnell SR (2011). Human NK cells proliferate and die in vivo more rapidly than T cells in healthy young and elderly adults. J Immunol.

[30] Hazeldine J, Hampson P, Lord JM (2012). Reduced release and binding of perforin at the immunological synapse underlies the age-related decline in natural killer cell cytotoxicity. Aging Cell.

[31] Lord JD, Shows DM (2017). Thiopurine use associated with reduced B and natural killer cells in inflammatory bowel disease. World J Gastroenterol.

[32] Steel AW, Mela CM, Lindsay JO, Gazzard BG, Goodier MR (2011). Increased proportion of CD16(+) NK cells in the colonic lamina propria of inflammatory bowel disease patients, but not after azathioprine treatment. Aliment Pharmacol Ther.

[33] Orange JS (2013). Natural killer cell deficiency. J Allergy Clin Immunol.

[34] Orange JS (2012). Unraveling human natural killer cell deficiency. J Clin Invest.

[35] Jiyad Z, Olsen CM, Burke MT, Isbel NM, Green AC (2016). Azathioprine and risk of skin Cancer in organ transplant recipients: systematic review and meta-analysis. Am J Transplant.

[36] Pasternak B, Svanström H, Schmiegelow K, Jess T, Hviid A (2013). Use of azathioprine and the risk of cancer in inflammatory bowel disease. Am J Epidemiol.

[37] Armstrong RG, West J, Card TR (2010). Risk of cancer in inflammatory bowel disease treated with azathioprine: a UK population-based case-control study. Am J Gastroenterol.

[38] Cottone M, Renna S (2009). IBD: incidence of HSV and HPV with azathioprine. Nat Rev Gastroenterol Hepatol.

[39] Seksik P (2009). Incidence of benign upper respiratory tract infections, HSV and HPV cutaneous infections in inflammatory bowel disease patients treated with azathioprine. Aliment Pharmacol Ther.

[40] Hellmich B, Flossmann O, Gross WL, Bacon P, Willem Cohen-Tervaert J, Guillevin L, Jayne D, Mahr A, Merkel PA, Raspe H, Scott DGI, Witter J, Yazici H, Luqmani RA, on behalf of the European Vasculitis Study Group (2007). EULAR recommendations for conducting clinical studies and/or clinical trials in systemic vasculitis: focus on anti-neutrophil cytoplasm antibody-associated vasculitis. Ann Rheum Dis.

[41] Nakazawa D, Masuda S, Tomaru U, Ishizu A (2019). Pathogenesis and therapeutic interventions for ANCA-associated vasculitis. Nat Rev Rheumatol.

