# Supplementary material for: Blood CD3-(CD56 or 16)+ natural killer cell distributions are heterogeneous in healthy adults and suppressed by azathioprine in patients with ANCA-associated vasculitides
Source: BMC Immunol. 2021 Apr 12;22:26. doi: 10.1186/s12865-021-00416-w (PMC8040212; doi:10.1186/s12865-021-00416-w)
Supplement: Supplementary file 1 — Additional file 1: Supplementary Fig. 1. The distribution of NK cell counts and percentages in AAV (one measurement/patient). HC, n = 120 healthy controls; Upper left, n = 93 first measurements of absolute NK cell counts in patients with ANCA-associated vasculitis (AAV). Upper right, n = 95 first measurements of NK cell percentages in blood lymphocytes. Lower row, n = 94 last measurements of NK counts and percentages, respectively. In each graph of this figure, the Kolmogorov-Smirnov tests were significant. Data derived from vasculitis center 1. [file 12865_2021_416_MOESM1_ESM.docx]

**Blood CD3-(CD56 or 16)+ natural killer cell distributions are heterogeneous in healthy adults and suppressed by azathioprine in patients with ANCA-associated vasculitides**

*Wolfgang Merkt^1^, MD, Ulrich Salzer^2^, MD, Jens Thiel^2^, MD, Ilona Jandova^2^, Raoul Bergner, MD^3^, Ana C. Venhoff^2^, PhD, Nils Venhoff^2^, MD*

**Supplementary material**


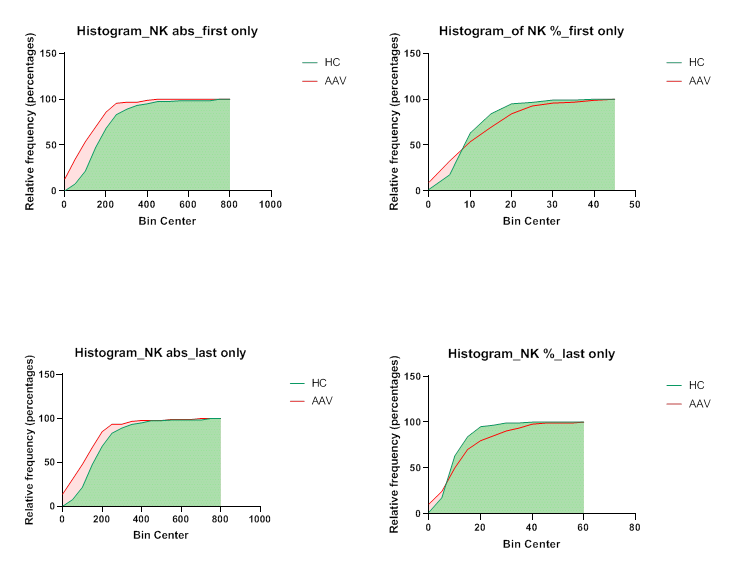


**Supplementary figure 1. The distribution of NK cell counts and percentages in AAV (one measurement/patient).** HC, n=120 healthy controls; Upper left, n=93 first measurements of absolute NK cell counts in patients with ANCA-associated vasculitis (AAV). Upper right, n=95 first measurements of NK cell percentages in blood lymphocytes. Lower row, n=94 last measurements of NK counts and percentages, respectively. In each graph of this figure, the Kolmogorov-Smirnov tests were significant. Data derived from vasculitis center 1.
